# Long-term degradation of U-values: A dataset of experimental evidence and energy performance consequences

**DOI:** 10.1016/j.dib.2025.111951

**Published:** 2025-08-05

**Authors:** H. Alkhatib, Omar K. Omar, B. Norton

**Affiliations:** a School of Mechanical Engineering, Technological University Dublin, Dublin, Ireland; b Dublin Energy Lab, Technological University Dublin, Dublin, Ireland; c MaREI, the SFI Centre for Energy, Climate and Marine, Cork, Ireland; d Mechanical Engineering Department, Al Hussein Technical University, Amman, Jordan; e IERC, International Energy Research Centre, Tyndall National Institute, University College Cork, Cork, Ireland

**Keywords:** U-value, Energy audit, Thermal performance, Building efficiency, Insulation

## Abstract

This dataset contains experimental U-value measurements collected over a three-year period (2022–2025) for various building envelope components, including front and back external walls, internal partitions, and roofs. The data reflects real-world degradation in thermal performance due to ageing and material changes. Each component is documented through raw CSV data, detailing the experimental setup, environmental conditions, and measurement duration. The dataset is intended to support research in building energy performance, retrofit evaluation, and the development of predictive models for thermal ageing in construction materials.

Specifications Table

**Table:** 

Subject	Energy
Specific subject area	Renewable Energy, Sustainability and the Environment
Type of data	Raw, Table and Figures
Data collection	The dataset was collected through in-situ measurements of heat flux and surface temperatures across multiple building envelope components, including external and internal walls, floors, roofs, and windows. Measurements were conducted over two campaigns in 2022 and 2025 using calibrated heat flux sensors and thermocouples, in accordance with ISO 9869–1:2014 standards. Data was recorded at 5-minute intervals over 7-day periods per component, allowing for accurate calculation of U-values under steady-state and near-steady-state conditions. Pre- and post-processing steps were applied to filter out unstable readings and ensure data reliability.
Data source location	All measurements were carried out on a test building located at Technological University Dublin's campus. The building featured representative envelope materials typical of residential and institutional construction. Sensors were installed on different orientations (north, south, internal, roof, floor) to capture environmental variability. Data was logged using a multi-channel data acquisition system, and environmental conditions were monitored using nearby weather station data.
Data accessibility	Alkhatib, Hani; Omar, Omar; Norton, Brian (2025), “Long-Term Degradation of U-Values: A Dataset of Experimental Evidence and Energy Performance Consequences”, Mendeley Data, V1, 10.17632/4kbb93bx32.1 Repository name: **Long-Term Degradation of U-Values: A Dataset of Experimental Evidence and Energy Performance Consequences.** Data identification number: **10.17632/4kbb93bx32.1** Direct URL to data: https://data.mendeley.com/datasets/4kbb93bx32/1 Instructions for accessing these data: **Non**
Related research article	**Non applicable.**

## 1 Value of the Data

• The dataset provides a detailed, longitudinal record of U-value degradation across multiple building envelope components, offering empirical evidence over a three-year period.
• It supports research on building energy performance, retrofit prioritization, and lifecycle thermal behaviour by providing real-world in-situ measurement data [1].
• The dataset highlights the significance of thermal ageing in envelope materials, showing measurable increases in U-values over time.
• By comparing data from different orientations and materials, it enables analysis of environmental exposure effects on thermal performance.
• This dataset is valuable for building physicists, energy modellers, and sustainability researchers focused on improving the accuracy of energy simulations and informing maintenance or renovation strategies.

## 2 Background

The thermal performance of building envelopes is an essential contributor to determining energy efficiency and occupant comfort in buildings. Thermal transmittance, or U-value, is central to this performance, which measures the rate of heat transfer through a building element per unit area per unit temperature difference between the interior and exterior environments. [[2], [3], [4], [5]]. Lower values of U-values represent better thermal resistance, which indicates better insulation and reduced energy loss. As buildings attempt to meet higher standards with respect to energy efficiency targets and net-zero goals, knowing and controlling U-values is essential to achieve long-term sustainable performance [[6], [7], [8], [9], [10]].

While most design practices and regulations consider U-values as static properties which are immutable once a building is built or refurbished, the truth is that building materials and components all change physically (and often chemically) over time as they weather and deteriorate. This may result in a deterioration of thermal resistance, creating higher U-values, and consequently requiring greater heating or cooling loads. The potential consequence of U-value degradation on energy consumption and occupant comfort is not extremely well-established. There remains a shortage of research on the changes to U-value over the longer-term (more than 1–2 years) in "real-world" circumstances. There is plenty of short-term data, limited long-term data, and even less long-term subjective data on different building types, climates and construction systems [[11], [12], [13]].

This study seeks to address this gap by investigating the degradation of U-values over time through empirical analysis. Specifically, it compares U-value measurements from the same building element taken three years apart in a controlled experimental setting [[14], [15], [16], [17], [18]].

## 3 Data Description

The dataset includes solar irradiance measurements collected using five calibrated pyranometers, with data recorded at 5-minute intervals over several days. It captures solar irradiance before and after calibration, along with temperature readings to ensure environmental monitoring. The calibration process followed ISO 9847 standards and involved calculating calibration factors for each pyranometer. To improve data accuracy, systematic checks were performed, and inconsistencies were identified and addressed. Pre- and post-calibration comparisons showed significant improvements in measurement precision, offering more reliable solar irradiance data for future solar energy applications. The dataset includes the pyranometer output in voltage (V) along with a calibration factor to convert the voltage into radiation measured in watts per square meter (W/m²). Different pyranometer models can use the same ISO standard for calibration.

The dataset consists of five component-specific CSV files, Front wall U value.csv, Back Wall U value.csv, Internal Wall U value.csv, Roof U value.csv, and Window U value.csv, as well as one TXT file, Weather.txt. Each CSV file contains time-series data collected at 5-minute intervals, including the following variables: Timestamp (DateTime), Indoor Surface Temperature ( °C) recorded via thermocouple, Indoor Air Temperature ( °C) from a separate sensor, Outdoor Surface Temperature ( °C) via thermocouple, and Outdoor Air Temperature ( °C). The dataset also includes Heat Flux (W/m²), which was derived from voltage signals using a calibration factor, and the corresponding U-Value (W/m²K), calculated based on these readings.

The U-values were calculated post hoc using the average method from ISO 9869–1:2014, applied after filtering out transient periods and ensuring data stability. Temperature and heat flux data were collected at 5-minute intervals using calibrated sensors connected to a multi-channel data logger. The Weather.txt file includes external temperature, irradiance, and humidity readings used for validation and filtering. Sample of raw data is shown in Table 1.

**Table 1:** Sample data table.

Timestamp	Heat Flux (W/m²)	Indoor Surface Temp (°C)	Indoor Air Temp (°C)	Outdoor Surface Temp (°C)	Outdoor Air Temp (°C)	U-Value (W/m²K)	Notes
09/05/2025 18:50	0.550	27.280	13.594	26.838	14.337	0.019	Raw data
09/05/2025 19:00	0.700	27.155	13.491	26.697	14.124	0.010	Raw data

## 4 Experimental Design, Materials and Methods

This study assessed the long-term degradation of thermal performance in building envelope elements by comparing U-values measured in 2022 and again in 2025. The experimental approach followed ISO 9869–1:2014 standards for in-situ thermal transmittance evaluation using heat flux and temperature sensors.

**Fig. 1:**
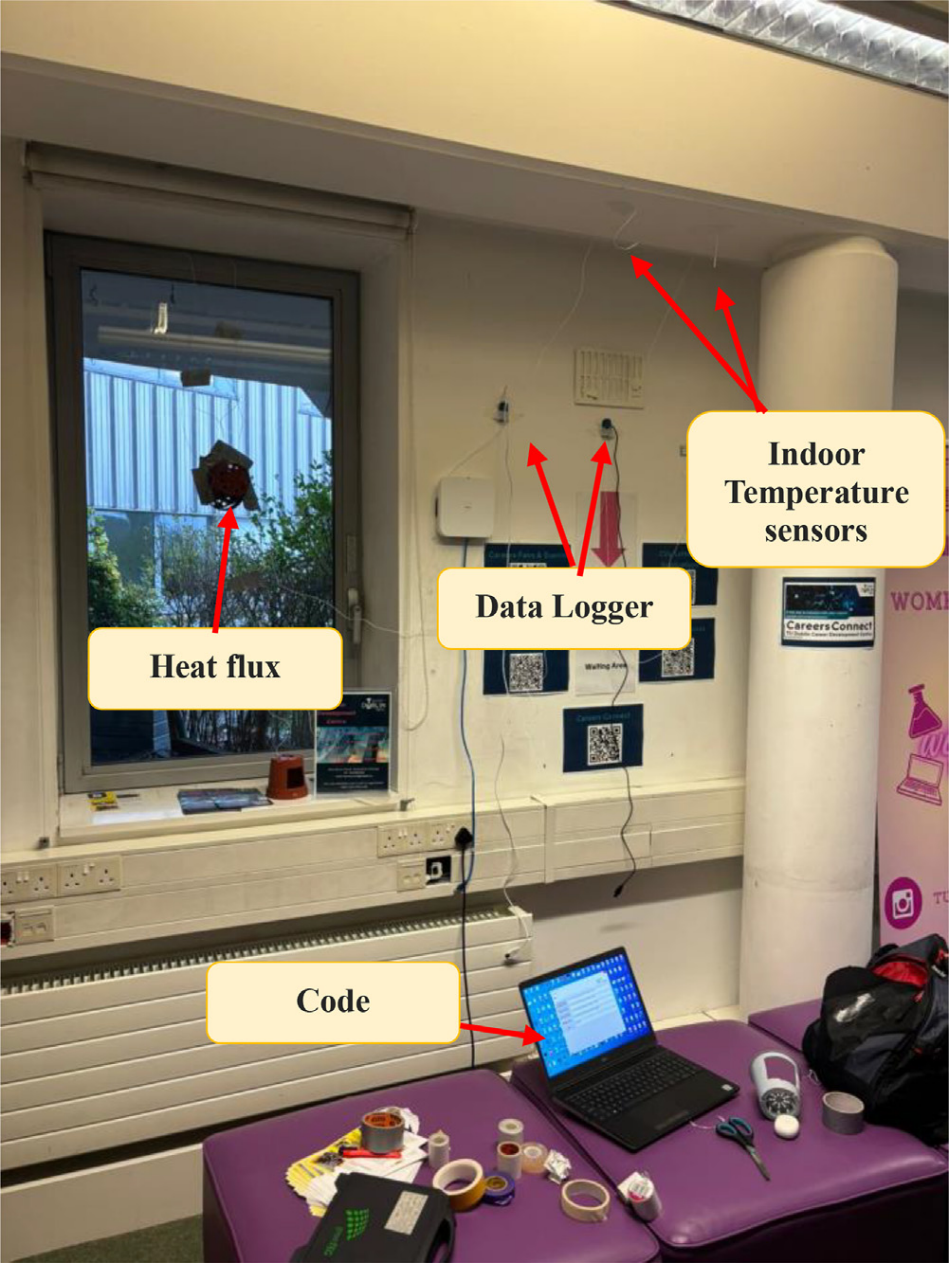
Experimental setup of the study.

As shown in Fig. 1, each test configuration involved a heat flux sensor mounted on the inner surface of the component under study, paired with indoor and outdoor thermocouples to capture surface and ambient temperatures. All sensors were connected to a DL2e data logger, which recorded data at 5-minute intervals continuously over a 7-day period for each element [19,20].

Six different envelope components were monitored: south-facing front external wall, north-facing back external wall, internal wall, roof, floor, and a double-glazed south-facing window. These elements were selected to represent varied orientations, exposures, and construction types. Measurements were repeated in the same locations in 2022 and 2025, ensuring consistency in setup and environmental context.

To ensure data quality, time-series data were cleaned to remove periods affected by transient internal loads (e.g., occupancy or appliance use), erratic sensor behavior, or sudden weather changes. The cleaning protocol was based on validated methodologies reported in prior literature and was essential to obtain stable U-value estimates under quasi-steady-state conditions. Post-processed data were used to compute U-values using the average method described in ISO 9869–1. The results, presented in Table 2 and Table 3, show clear increases in U-values across all components over the three-year interval, with corresponding graphs illustrating heat flux and temperature variations over time [21].

**Table 2:** Measured overall heat loss coefficients for primary building elements in 2022 and 2025 (W/(m²K)).

**Table 3:** Measured overall heat loss coefficients for primary building elements over three years and percentage difference.

Building Element	Orientation	Duration of test (h)	Overall heat loss coefficient (2022) (W/(m²K))	Overall heat loss coefficient (2025) (W/(m²K))	% Difference
**Front External wall**	South	168	1.45	1.81	25
**Back External wall**	North	168	0.26	0.38	46
**Internal Wall**	Internal	168	0.45	0.97	116
**Roof**	Roof	168	0.13	0.36	177
**Window**	South	168	2.98	3.36	13
**Floor**	Floor	168	0.57	0.74	30

Data cleaning was performed to exclude unstable readings and transient conditions that could compromise the accuracy of the U-value calculations. Specifically, data points were removed when the indoor–outdoor temperature difference was less than 5 °C, when heat flux values fluctuated by more than ±10 % over a 15-minute period, or when indoor air temperature changed by more than 1 °C within 10 min. These criteria were applied consistently across all test cycles to ensure that only steady-state or quasi-steady-state data were retained for analysis. Data points collected during known occupancy or appliance use were also filtered using time logs. On average, 8–12 % of data points per test cycle were removed through this filtering process to ensure steady-state or quasi-steady-state conditions as required by ISO 9869–1.

Measurements were conducted under ambient, real-world conditions. Internal spaces were generally unconditioned and left in a passive state, except for occasional occupancy-related heat gains. No active heating or cooling systems were in operation during measurement periods. This setup was chosen to reflect realistic envelope performance without artificial boundary control.

The sensor layout followed ISO 9869–1:2014 recommendations. For each component, a heat flux sensor was mounted on the internal surface, while thermocouples were placed on both indoor and outdoor surfaces as well as in the adjacent room air. Sensor positions were chosen to avoid thermal bridges and edges, ensuring measurement in the most thermally representative area.

### 4.1 Equipment

Table 4 shows the accuracy, range and measuring interval of two instruments used to measure the U-value of the building fabric. Each U-value kit consists of two temperature sensors, a heat flux meter, and a data logger. These components work together to collect precise data on thermal performance. The data logger records the measurements, while a PC is used to write the code for data logging and to set the recording interval. The U-value calibration factor was also determined using specialized software. This setup ensures accurate and reliable measurement of the building’s thermal properties, essential for a comprehensive energy audit [22].

**Table 4:** Instrument used to measure the U-value.

## Limitations

‘Not applicable’.

## Ethics Statement

**All authors** have read and follow the ethical requirements for publication in Data in Brief and confirming that the current work does not involve human subjects, animal experiments, or any data collected from social media platforms.

## CRediT Author Statement

**Hani Alkhatib:** Conceptualization, Methodology, Investigation, Visualization, Writing – original draft, Writing – review & editing; **Omar K. Omar:** Conceptualization, Methodology, Supervision, Validation, Software; **Brian Norton:** Supervision, Methodology, Validation, Conceptualization, Methodology.

## Data Availability

Mendeley DataLong-Term Degradation of U-Values: A Dataset of Experimental Evidence and Energy Performance Consequences (Original data)
